# Customized 3D-printed stackable cell culture inserts tailored with bioactive membranes

**DOI:** 10.1038/s41598-022-07739-7

**Published:** 2022-03-07

**Authors:** Asli Aybike Dogan, Martin Dufva

**Affiliations:** Department of Health Technology, Technical University of Denmark, 2800 Kgs. Lyngby, Denmark

**Keywords:** Lab-on-a-chip, Biomedical materials, Assay systems

## Abstract

There is a high demand in various fields to develop complex cell cultures. Apart from titer plates, Transwell inserts are the most popular device because they are commercially available, easy to use, and versatile. While Transwell inserts are standardized, there are potential gains to customize inserts in terms of the number of layers, height between the layers and the size and composition of the bioactive membrane. To demonstrate such customization, we present a small library of 3D-printed inserts and a robust method to functionalize the inserts with hydrogel and synthetic membrane materials. The library consists of 24- to 96-well sized inserts as whole plates, strips, and singlets. The density of cultures (the number of wells per plate) and the number of layers was decided by the wall thickness, the capillary forces between the layers and the ability to support fluid operations. The highest density for a two-layer culture was 48-well plate format because the corresponding 96-well format could not support fluidic operations. The bottom apertures were functionalized with hydrogels using a new high-throughput dip-casting technique. This yielded well-defined hydrogel membranes in the apertures with a thickness of about 500 µm and a %CV (coefficient of variance) of < 10%. Consistent intestine barrier was formed on the gelatin over 3-weeks period. Furthermore, mouse intestinal organoid development was compared on hydrogel and synthetic filters glued to the bottom of the 3D-printed inserts. Condensation was most pronounced in inserts with filters followed by the gelatin membrane and the control, which were organoids cultured at the bottom of a titer plate well. This showed that the bottom of an insert should be chosen based on the application. All the inserts were fabricated using an easy-to-use stereolithography (SLA) printer commonly used for dentistry and surgical applications. Therefore, on demand printing of the customized inserts is realistic in many laboratory settings.

## Introduction

In vitro tissue/organ modelling is an increasingly popular field for tissue engineering and drug development. For instance, organ systems have been employed to model pharmacokinetics and dynamics^1–4^, first-pass metabolism^5^, and uteritis colitis^6^. The field has been dominated by microfluidics chips due to their excellent mass transfer characteristics. However, microfluidics chips are often challenging to use and not easily scalable in terms of the number of experiments that can run in parallel. Transwell-type of inserts for in vitro organ models have therefore been explored^5,7^.

Transwell hanging inserts have been extensively used to recreate single barrier tissues like the intestine, skin, and blood–brain-barrier. They are also used to interconnect tissues by co-culturing multiple cell lines or creating 3D tissue such as organoid cultures with hydrogels^8–10^. These inserts are standardized with permeable supports with microporous synthetic membranes^11^ such as polycarbonate (PC), polyester (PET), polytetrafluoroethylene (PTFE)*.* The pore sizes range from 0.4 to 8 µm, the thickness is between 10 and 30 µm, and the porosity is 10–15%. The drawbacks of commercial Transwell inserts are the lack of flexibility to choose the material and size of the bioactive cell culture area. Another limitation is that Transwell inserts provide only one layer.

Custom-made, three-layer 3D-printed insert plates for interconnecting different tissues in the 12-well format have recently been demonstrated^5^. In contrast to the relatively complex and expensive fabrication process of culture devices such as injection molding^12^, 3D printing is superior due to its flexibility to create customized inserts rapidly and cost-efficiently for low-volume, lab-scale production. Hanging inserts are relatively large that are well within the bounds of the performance of even low-cost 3D printers. The low-cost extrusion-based filament printers could potentially be used to manufacture inserts. However, even though polymers such as native polylactic acids (PLA) are biocompatible^13^, printing smaller features like holes with this technique is challenging because the resolution is in several hundreds of µm. It is also unclear if the insert walls are completely sealed with filament printing which is necessary to block liquid or the electrical current when analyzing the cell barriers. The SLA and projection printers (e.g., digital light processing (DLP) printers) have better resolution and provide liquid-proof prints^14^. In these printers, light is utilized to crosslink a photo-curable resin that is biocompatible for cell culture applications^14,15^. Some resins such as Dental LT from Formlabs conform to biocompatibility class IIa because the prints are intended for dentistry. Even though dental resins are biocompatible, they do not support cell attachment^5^. This property allows for defining the bioactive area with a filter or a hydrogel to resize and shape the cell culture area according to experimental needs.

Hydrogel scaffolds have high-water absorption capacity due to their permeable/porous structures. They are widely used as ECM-mimetic biomaterials and are shaped by film/membrane formation^5,16^, molding^17,18^, or 3D bioprinting^19,20^. Hydrogels as a membrane (here, we define all membranes as the material closing the bottom of an insert) make it possible to utilize soft growth matrices instead of the rigid filter membranes used in Transwell inserts. Cells sense the stiffness of their growth matrices^21,22^, and the intrinsic biodegradability of hydrogels is considered a crucial feature during tissue regeneration^23^. Various natural, synthetic, and hybrid biomaterials such as Poly (ethylene glycol) (PEG)^24^, silk^25^, Matrigel^8^, decellularized tissues^26^, and gelatin^5,16,27–29^ have been explored in the literature. An advantage of hydrogels is that they are often transparent, which is not always the case for filters. Transparency is a major feature for microscopy.

From our previous work^5^, we have identified three district challenges addressed here. Firstly, the 12-well plates are not suitable for high-throughput screening (HTS), where the 96-well plate format dominates due to its lower reagent usage. Secondly, the method for functionalizing the insert with a hydrogel membrane must be simplified if 96-well plates are used. Thirdly, the previous hydrogels were 1–3 mm thick, resulting in slow mass transport through the membrane. Hydrogels have been casted to obtain defined thicknesses which requires a custom-made apparatus^5^. Scaling this approach to 96-well will be difficult because it requires exchangeable surfaces that repel gelatin, such as parafilm^5^ or pristine PDMS.

To address these challenges, a dip-casting method to form hydrogel membranes was developed and validated. The procedure reliably created 500 µm thin gelatin membranes. We also challenged the 3D printing technology to scale the 12-well inserts to 24-, 48- and 96-well plate formats as strips, singlets, and multiple stacked layers, to investigate physical or mechanical limitations of stacking. We also described a method to assemble synthetic filters to the insert bottom. We investigated the biocompatibility of 3D-printed custom inserts on mouse intestinal organoids and epithelium barriers.

## Results

### Physical limits of stacked inserts

**Figure 1:**
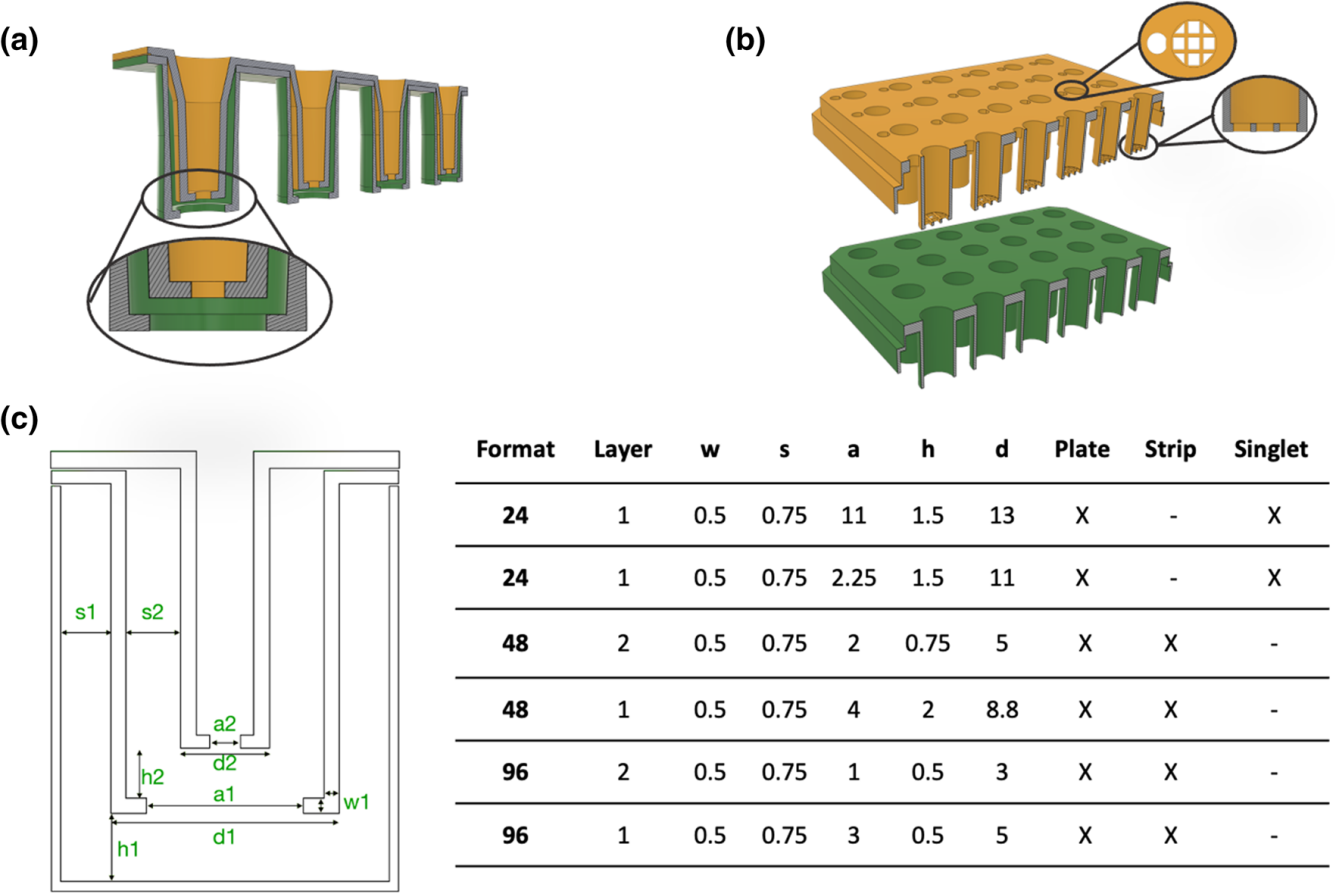
Representative illustrations of 3D-printed inserts. (**a**) Stacked (layering) insert approach. (**b**) 24-Well format insert plates. **(c**) Dimensions of introduced 3D-printed inserts and representative examples of singlet, strip, and plate format inserts in the online library (*w* width, *s* distance between layer walls, *a* aperture, *h* height from the well/insert below, *d* diameter; all dimensions in mm.)

24- to 96-well inserts were designed as full plates, strips, and singlets (Fig. 1 and Supplementary Fig. S1). The design followed the previous described modular vertical stacking of cell culture inserts where insert slide into each other^5^. We considered the wall thickness, the wall-to-wall distance, and the number of layers that could be stacked in the respective well format. The distance between the bottoms of the respective insert layers was not optimized but kept to 500–750 µm. Generally, the insert closest to the bottom of the well was 1–2 mm from the bottom of the well to enable microscopy using a long working distance objective without moving the inserts stacks from the plate. The 24-well format had access ports for medium sampling and TEER measurements without disturbing the cultures (Fig. 1b). Due to space restraints, access ports were not included in the 48- or 96-well formats.

As the wall of each insert layer fills in the well, it is limited how many layers a specific well size can hold. We focused on 2-layer stacks for 96- and 48-well plate formats (Fig. 1a) to investigate how small stackable inserts could be and yet be functional. We aimed to minimize design dimensions to be able to stack multiple layers by limiting capillary effects and bubble formations due to trapped air during the stacking of the layers. The wall thickness of each printed insert was 545 µm ± 34 µm (mean ± SD; n = 36) with ± 80 µm tolerance to the design dimension (500 µm). 250 µm thin walls were not rigid, and inserts got damaged during stacking (data not shown). The distance between the two walls in the respective stacked insert was 757 µm ± 22 µm (mean ± SD; n = 6; with ± 30 µm tolerance to the design dimension: 750 µm). Smaller wall distances made stacking insert layers difficult, especially regarding entrapment of bubbles and capillary effects. Thus, each insert layer occupies about 2.5 mm in diameter (2*750 + 2*500 µm) plus the inner diameter, which limits the number of layers in a well. Both 48- and 96-well plate sizes could hold two stacked inserts, but the topmost insert in the 96-well plates was as narrow as a well in 384-well plates (Ø_inner_ = 2 mm, Fig. 1c). Such narrow wells are easy to operate manually when the tip of the pipette is hitting the insert bottom before ejecting the liquid. This mode of operation is not possible because that would destroy cell layers or the hydrogel membranes. Therefore, the 96-well format could not hold two layers. The bottom-most insert in the 96-well formats was by contrast 5 mm in diameter, which we consider to be a minimum usable inner diameter.

The bottom of the insert had one large hole, an array (Fig. 1b) or a pattern of smaller holes (Supplementary Fig. S2). One large hole was used for gluing or adhering rigid polymeric filter membranes to the insert (Supplementary Fig. S1).

### Functionalization of the inserts with filter membranes

Hydrophilic PTFE membranes were used to modify the bottom of the inserts with a microporous filter membrane. The PTFE membrane was glued using the Dental LT resin to 3D-printed 96-, 48- and 24-well plate inserts with one large aperture in the bottom. Initial trials included applying a thin layer of uncured Dental LT to the rim of the bottom. However, we often observed that the resin absorbed into the filter, which resulted in blockage after UV crosslinking (Supplementary Fig. S1a-top left panel). This was especially troublesome for small formats such as 96-wells. An improved gluing process was developed where the resin was applied to the sides of the 3D-printed insert (Supplementary Fig. S1b). The PTFE membrane was placed on the bottom and secured with a ring slipped over the bottom of the insert. The ring was pressing the filter towards the sides of the insert. The excess resin was absorbed in the filters on the sides of the inserts rather than the bottom, which minimized the blockage of the filter in the aperture (Supplementary Fig. S1a-top right panel). The PTFE membrane could not be removed without destroying it (data not shown), which indicated a tight seal.

### Functionalization of the inserts with hydrogel membranes

**Figure 2:**
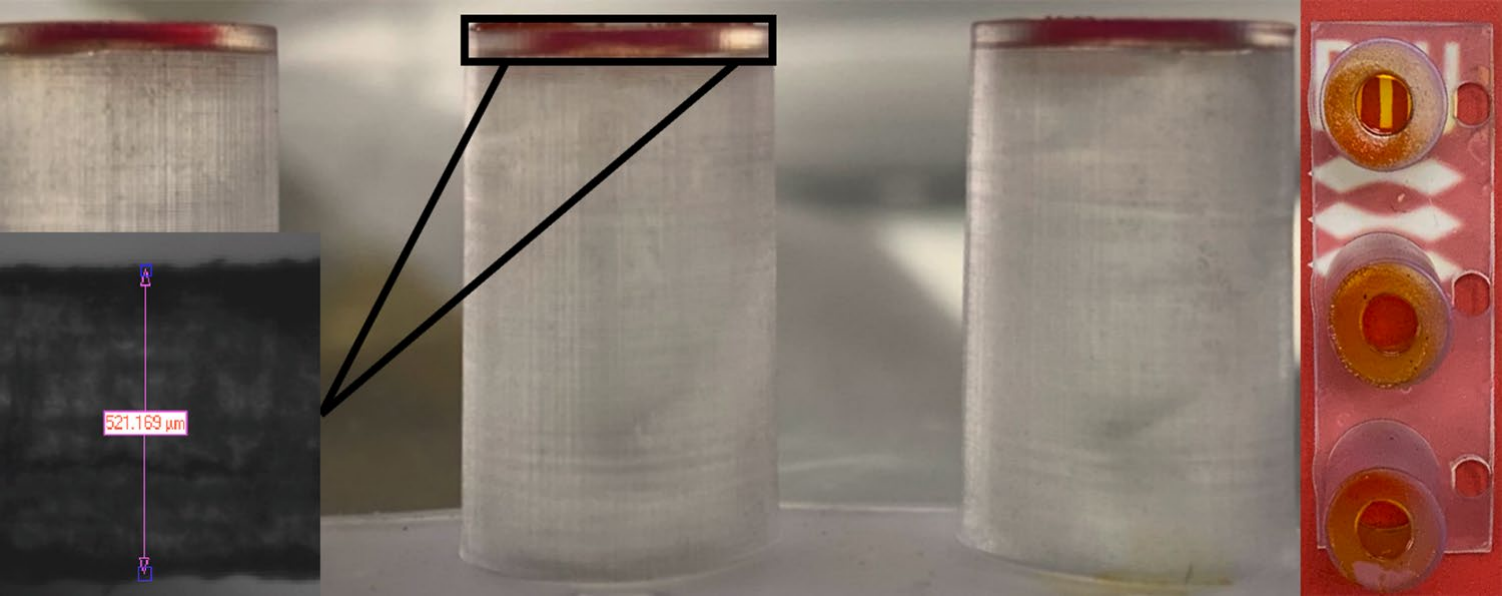
3D-printed Gelatin-inserts (Ø: d = 8.8 mm; a = 4 mm); dip-cast with red food dye colored gelatin membrane. *Left* The thickness of the 3D-printed material at the bottom, 521 µm) was measured with a 6.5-fold zoom lens USB 3 camera of contact angle device (Dataphysics, Germany). *Right* Transparency of a dry gelatin membrane.

A quick and scalable hydrogel functionalization method was developed and characterized based on a tool-free casting procedure^16^. The viscosity was controlled by keeping gelatin (without mTG) at 37 °C. The device was dipped a few mm into the gelatin solution, and the immersion time was standardized to 5 s. The devices were subsequently gently removed (Supplementary Fig. S3). Excess gels solution was removed by sliding the bottom of the insert against a warm sterile petri dish. The procedure ensured that only the apertures were filled with the hydrogels (Fig. 2). This functionalization method was also compatible with agarose and soy-based hydrogels (data not shown).

Irreversible crosslinking was achieved using a two-step protocol. Firstly, the gelatin membranes were reversibly physically crosslinked by reducing the temperature to 4 °C. The gelatin membranes were subsequently irreversibly chemically crosslinked by adding a solution of mTG^29^. The formed membranes could be used immediately, stored in a refrigerator, or even dried and stored at room temperature for later use. A dried membrane rehydrates quickly and supports cell cultures (e.g., Caco-2, data not shown).

**Figure 3:**
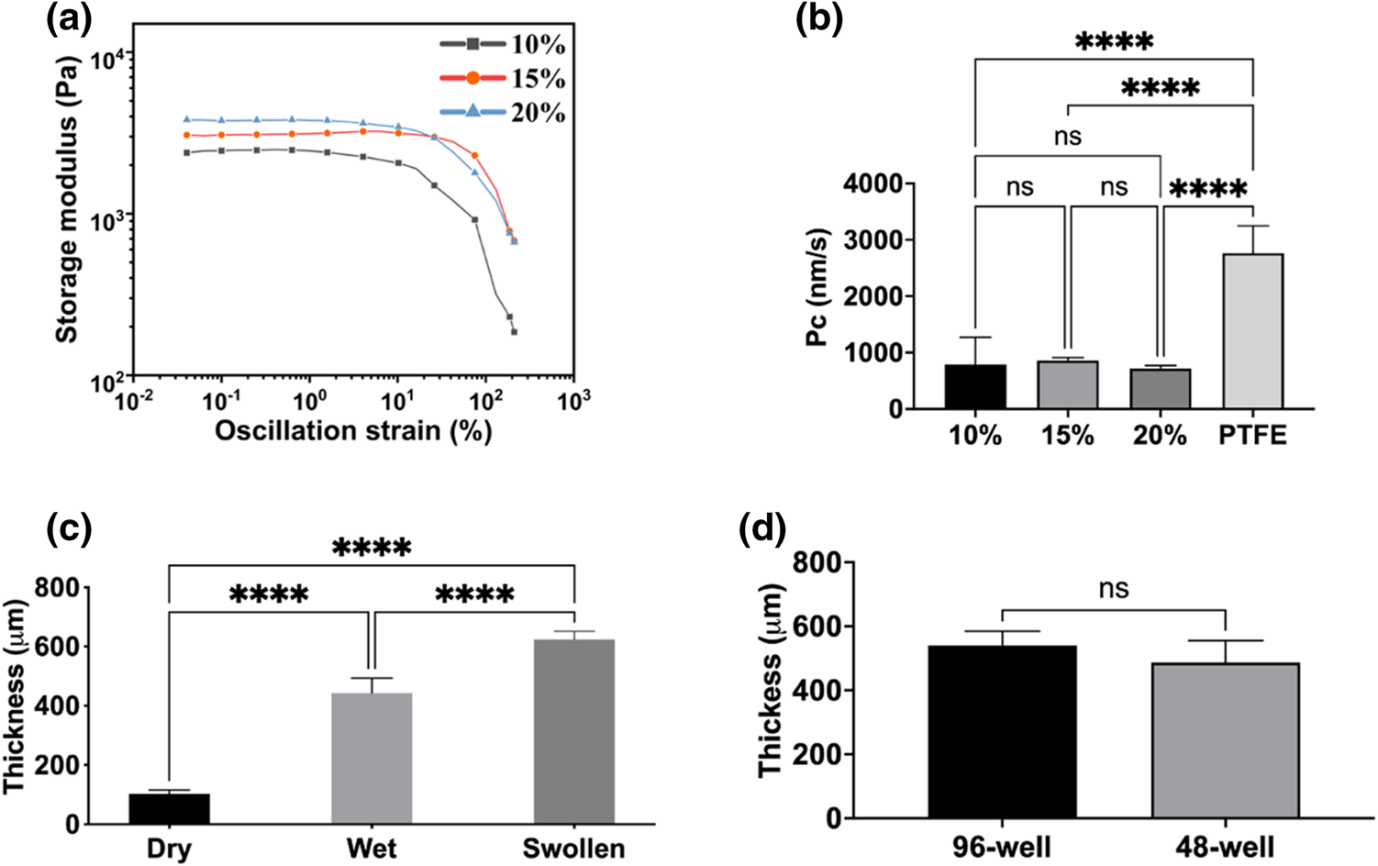
Determination of mechanical properties of 10% w/v (10%), 15% w/v (15%), 20% w/v (20%) gelatin membranes. (**a**) Analysis of storage and loss modulus of each membrane under applied oscillatory compression. (**b**) Comparison of 20 µM Fluorescein sodium salt dye transport through each membrane assembled to 96-well plate format 3D-printed strip insert wells (10–20%: n = 4; PTFE: n = 7). Insert well aperture surface area for transport is 0.05 cm^2^. (**c**) Determination of thickness changes of 15% (w/v) Gelatin membranes assembled to 48-well format (Ø: d = 8.8 mm; a = 4 mm); wet (n = 5), swollen (n = 5), and dry (n = 5). (**d**) Thicknesses measurement of 15% (w/v) gelatin membranes (n = 5) via fluorescence intensity analysis. Error bars indicate mean ± SD. Statistical significance: ns = P > 0.05, ****P < 0.0001.

The range of gelatin concentrations that could be used in the process was investigated. 2.5% and 5% gelatin solutions did not give consistent results, and damaged membranes were often observed after the dip-casting and crosslinking procedures. 10% w/v (10%), 15% w/v (15%), 20% w/v (20%) gelatin concentrations resulted in 25–30% failures which was acceptable. Functionalizing apertures larger than 4 mm typically increased the failure rate. We estimated the respective compositions’ elasticity and stiffness by analyzing rheology at 37 °C. The calculated shear/complex modulus (G* =  ~ 2.48, ~ 3.12, ~ 3.81) and Young’s modulus (E =  ~ 7.44, ~ 9.36, and ~ 11.44 kPa) for 10, 15 and 20% gelatin respectively (Fig. 3a).

The transport characteristics of hydrogels (10%, 15%, 20%) were compared with hydrophilic PTFE membrane (Fig. 3b) using a standard curve of fluorescein salt as a reference (Supplementary Fig. S4-right panel). As mass transport is a function of gel thickness, these results also indirectly measure the consistency of the formed membranes. Each hydrogel membrane had no significant difference in the mass transport of Fluorescein salt dye, while they were significantly slower than the hydrophilic PTFE membrane (P < 0.0001). The %CV (coefficient of variance) was 16% (PTFE), 6.7% (10%), 6% (15%), 13% (20%), showing that the variance of the mass transport through dip-cast membranes was low.

The thicknesses of membranes were measured using two different methods. 48-well insert (Ø: d = 8.8 mm, a = 4 mm) was chosen as it allowed for using a micrometer screw to measure the membrane thickness in dry, wet, and swollen status. The thicknesses for casted and crosslinked hydrogel membranes were 440 ± 50 µm (mean ± SD). The thicknesses for membranes swollen in PBS for 1 h at 37 °C were 620 ± 27 µm (mean ± SD). The thicknesses for membranes dehydrated overnight at room temperature were 103 ± 12.5 µm (mean ± SD). Gelatin membranes swelled approximately 6 times from the dry phase (Fig. 3c). As the second method for measuring the thickness, 1 µg/mL Fluorescein sodium salt was added to the gelatin solution. Fluorescence membranes were dip-casted, and the fluorescence intensity was measured using a microscope. The thickness in µm was obtained using a standard curve of fluorescence labelled hydrogels with known heights (from 150 to 900 µm) (Supplementary Fig. S4-left panel). No significant differences were found between the thicknesses of membranes of 96-well inserts 540 ± 44 µm, and 48-well inserts 487 ± 68 µm (mean ± SD, p = 0.4244) (Fig. 3d). These results confirmed the results obtained with the micrometer screw method. (Fig. 3c).

All gelatin concentrations had similar mechanical properties, thickness, and transport properties. However, the different concentrations functioned differently in the dip-casting process where 10% (w/v) gelatin solution had lower viscosity but formed more fragile membranes. In comparison, 20% (w/v) gelatin had higher viscosity which made the dip-casting step more difficult. 15% (w/v) gelatin membrane composition was, therefore, a good compromise.

### Biological validation of the dipping approach using transport studies

**Figure 4:**
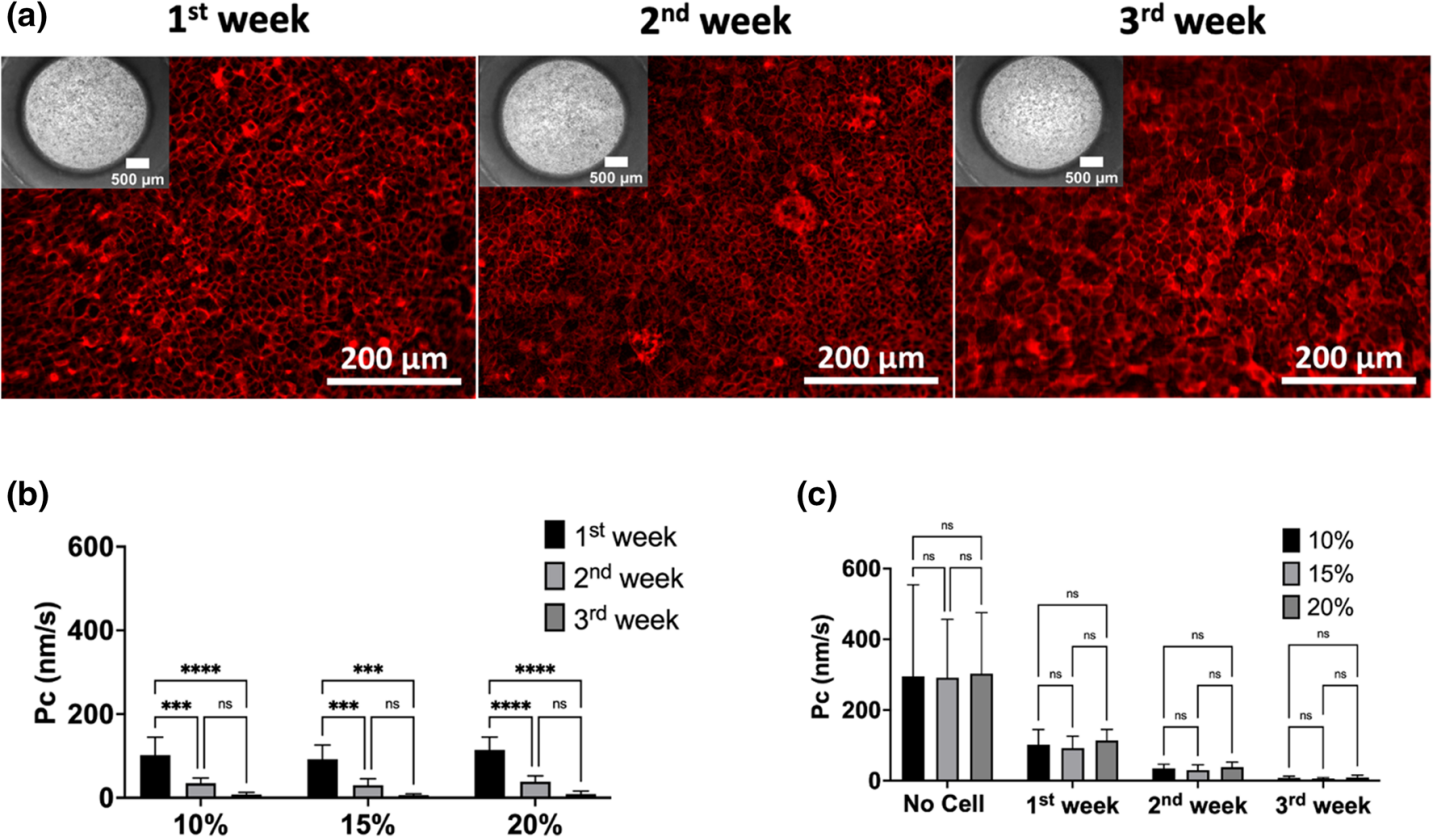
Biological validation of 96-well inserts. (**a**) Growth of Caco-2 cells on the 15% (w/v) gelatin membranes over three weeks. The 10% and 20% cultures had identical morphologies, so only the 15% cultures are displayed. Insert apertures at the bottom are shown at upper panels of fluorescence images (the magnification: 4x; scale bars: 500 µm), and the F-actin (red) stain of the cells on the hydrogel membranes (scale bars: 200 µm; the magnification was 20x). (**b**) Permeability coefficient (mean + SD) of lucifer yellow across Caco-2 cells on the 10%, 15%, 20% (w/v) gelatin membrane assembled inserts (n ≥ 3) and (**c**) comparison of Pc values of empty hydrogel, 10%, 15%, 20% membrane assembled inserts (n ≥ 5). Error bars indicate mean S.D. statistical significance: ns = P > 0.05, ****P < 0.001, ****P < 0.0001.

The dip-casting process was tested for sustaining long-term cell growth using 96-well inserts (Ø: a = 3 mm). Caco-2 cells were cultured and differentiated on the 10%, 15%, 20% gelatin membranes for three weeks to create intestinal barrier models. Caco-2 cells displayed cobblestone morphologies on all membrane conditions (Fig. 4a). Caco-2 cells were confluent in week 1 and kept their stability on the membranes during the 3-week incubation (Fig. 4a). F-actin (phalloidin staining) was expressed at the cell–cell border and is an indicator for tight interactions of cells^30^ (Fig. 4a). There was no apparent difference in morphology of cells grown on the respective hydrogels with the different gelatin concentrations (Supplementary Fig. S5).

TEER using chopstick electrodes could not be performed due to lack of space (see above) in the 48- and 96- well inserts. Instead, lucifer yellow (LY) transport was performed to assess barrier function as the permeability rates (Pc) correlate with barrier function^5,31,32^. Using an LY standard curve (Supplementary Fig. S6), transport rates were estimated to be on average 4.1 ± 2.5, 1.5 ± 3.4, 0.5 ± 1.0 nm/s, respectively (mean ± SD) in week 3 (Fig. 4b). By contrast, a gel without cells showed transport rates of 261 ± 113, 260 ± 74, 270 ± 79 nm/s, respectively, over the three different hydrogels (mean ± SD, Fig. 4c). It is considered that Pc ≤ 12 nm/s describes a well-established intestinal barrier function of Caco-2 cells^5,31,32^.

### Biocompatibility of the inserts for mouse intestinal organoid culture

**Figure 5:**
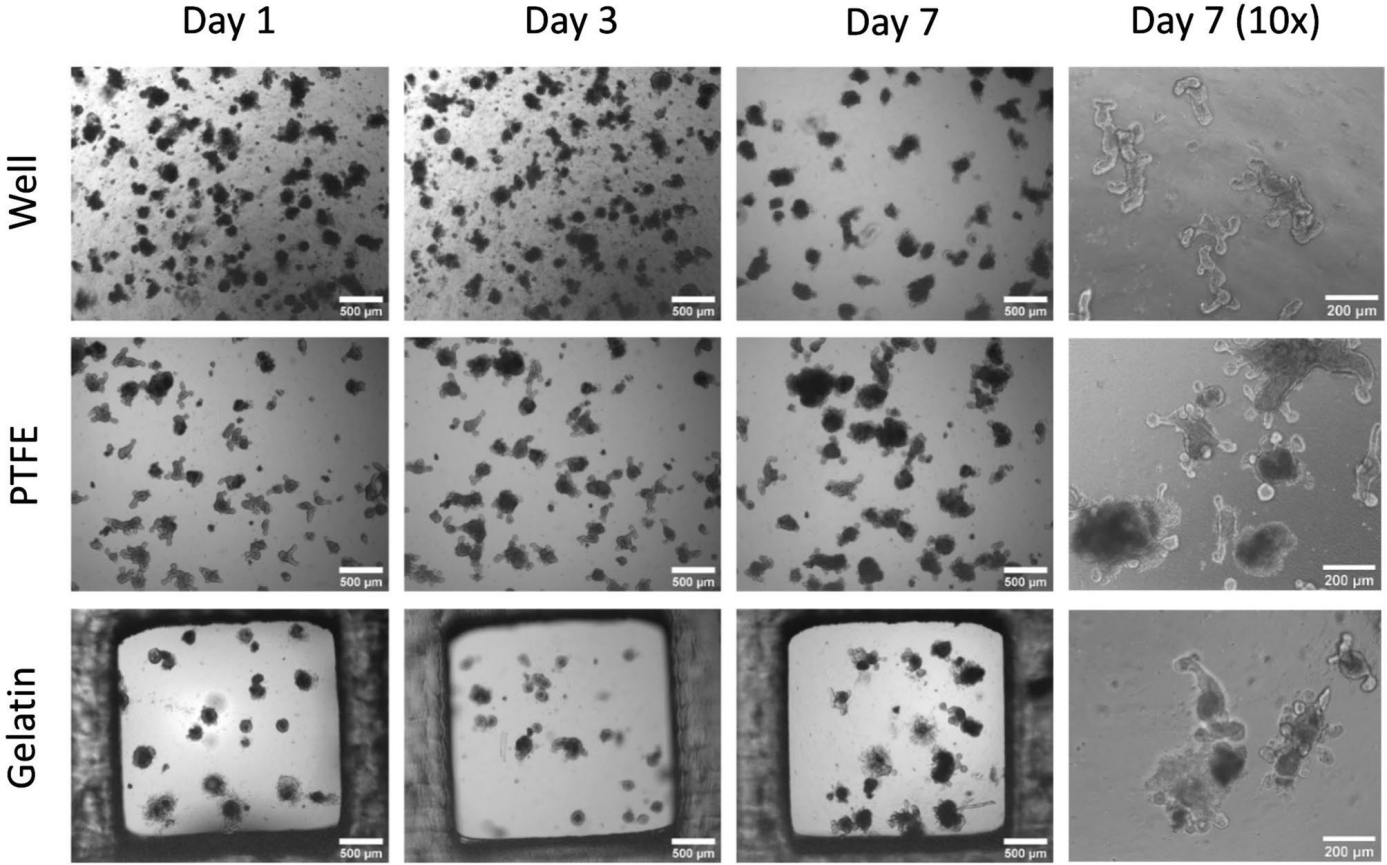
Representative light microscopy images of mouse intestinal organoids in Matrigel dome on hydrophilic PTFE filters and Gelatin membrane. Images (n = 3) were taken during their culture for 7 days in the insert plate wells with a light microscope (scalebar 500 µm for 4 × magnification; scalebar: 200 µm for the 10 × magnification.

The biocompatibility of the inserts was studied by culturing mouse intestinal organoids in 3D Matrigel dome culture on the well plate (control), hanging insert plates with filter (PTFE) and gelatin membranes, respectively (Fig. 5). Despite the same initial cell density and Matrigel contents, the culture behaved differently from each other. The organoids on filters and gelatin showed larger aggregates with very few smaller clusters at day one, while culture in wells displayed smaller organoids and many smaller clusters (Fig. 5). The organoids are surrounded by single cells on gelatin but not on the other culture conditions.

**Figure 6:**
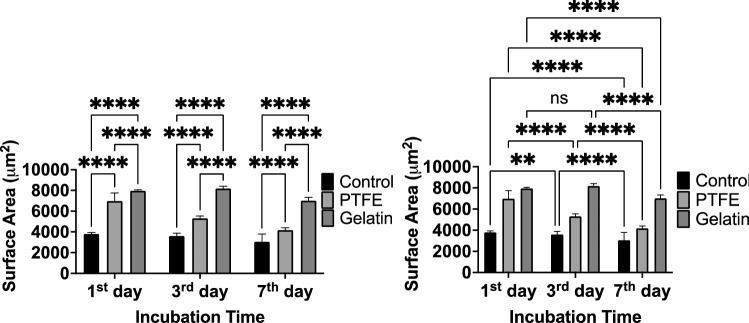
The surface area of organoids during 7 days of culture. *Left* Comparison of size changes of organoids depending on the surface substrate. *Right* Comparison of size changes of organoids during the incubation time. Error bars indicate mean S.D. statistical significance: ns = P > 0.05, **P < 0.01, ****P < 0.0001.

On day 3, the well cultures still had many smaller clusters while organoids began to get compact on the filter but not on gelatin. During the 7-day incubation period, surface areas of organoids were significantly reduced (20% for control, 40% for filter insert, 12% for gelatin inserts) (Fig. 6-Right). The condensation of organoids initiates villus morphogenesis is essential for the maturation of intestinal tissue^33^. Organoids on the gelatin membrane showed approximately two times higher surface area than culture on the filter membrane (Fig. 6-Left) after 7 days. Both insert groups showed a significantly larger surface area of organoids (filter-insert: 4170 µm^2^; gelatin-insert: 7000 µm^2^) compared to the control group (3000 µm^2^) after 7-day incubation. Nevertheless, differentiation and maturation indicators such as extruding cells into the central lumen structure, multiple buds, and multi-crypt structures were observed in all conditions (Fig. 5). On day 10, Live & Dead staining was performed, and it showed only a few dead cells in the buds in filter-insert groups (Supplementary Fig. S7). By contrast, more cell death and dispersion were observed in the gelatin-insert group. In conclusion, filter-inserts provided a more suitable microenvironment than gelatin-inserts or the well for 3D-cultured mouse intestinal organoids.

Taken together, the Dental LT material is biocompatible for a range of cells lines and processes. The results suggest that Dental LT is biocompatible and compatible with complex processes like organoid formation, which is more demanding than the maturation of a Caco-2 layer which previously has been described^5^.

## Discussion

Here, we present a small library of inserts that challenge the physical limits of 3D printing, capillary effects, and liquid handling. While 96-well plate format was achievable, only one layer could be used due to the difficulties of liquid handling. 48-well plates could, by contrast, host two usable layers of inserts. The dip-casting method was robust and yielded hydrogel membranes in the range of around 500 µm thicknesses, which correlated with the wall thickness of the 3D-printed insert. The dip-casting process resulted in < 10% variance in thickness. The biocompatibility was tested with mouse intestinal organoids. Organoids formed better on filters than at the bottom of a titer plate well or inserts functionalized with gelatin hydrogels. This indicates that the bottom of the insert should be chosen to match the requirements of the specific experiments.

The membrane-forming method was based on the dip-casting procedure (Supplementary Fig. S3), a simple, quick, and low-cost technique that relies on a range of physical and chemical parameters. Casting between two well-defined molds is less dependent on such parameters but requires a complex apparatus and non-sticky molds. During the dip-casting procedure, the gelatin liquid film is formed due to the adhesion of the gelatin liquid to the rim of the 3D-printed material. The surface tension and other physical properties such as adhesion and cohesive forces^34^ contribute to filling the holes. Although there is a significant failure rate (25–30%), the inserts that were successfully functionalized had very consistent properties of the gelatin membrane in terms of transport (Fig. 3) and thicknesses (Fig. 3c,d) and support for intestine barriers (Fig. 4). However, the process is dependent on many parameters, and just a few have been addressed here. Parameters such as immersion time/depth, withdrawal speed, removal of excess gelatin, number of dip-casting cycles, density/viscosity of the hydrogels, evaporation/crosslinking conditions of the solution and material characteristics of the insert may influence the membrane formation. Mixing mTG solution with gelatin creates a time-dependent increase of the viscocity^5,16^. To avoid that, we modified the protocol to control the viscosity. The dip-casting was done at 37 °C, a temperature in which pure gelatin keeps the viscosity for a long time. After the thermal setting of the gelatin at 4 °C, it was subsequently irreversibly crosslinked by adding a solution of mTG. The incubation with mTG was at 37 °C, which would melt the gelatin again. The small volume of mTG solution on top of the cold gelatin membrane likely initiated irreversible crosslinking of the gelatin, especially at the membrane surface, suppressing the dissolution of the gelatin in the mTG solution. Pressing and dragging the inserts toward a sterile, warm petri dish were used after dipping to remove excess gelatin, which would increase the variance of the obtained thickness. However, this process created failures with low-dense (≤ 5% w/v) gelatin solutions or large apertures, suggesting that the soluble gel needs to have a certain viscosity to support the process. Therefore, smaller apertures in the insert bottom were designed to protect the gel from physical strains of the casting process (Supplementary Fig. S2).

The mouse intestinal organoids performed better on filters than gelatin and the wells (Fig. 5). This observation correlated with mass transfer to and away from the culture (Fig. 3b). Presumably, the cultures are fed best through the filter, followed by the gelatin and the bottom of the well. The latter is used in dome culture approaches^35^. The result with intestinal organoids showed that a filter sometimes is preferable. However, a filter does not have any self-adhesive properties and therefore needs to be glued or thermally sealed. Gluing with Dental LT was possible but was not trivial as it was quite easy for the filter to absorb the resin. This resulted in clogging of the pores upon UV crosslinking (Supplementary Fig. S1a-bottom panel). The absorption was minimized by applying the resin on the sides of the insert (Supplementary Fig. S1b). This manual process is, however, cumbersome. A photomask defining the gluing areas followed by a rinse to remove the monomers could be used as an alternative. A similar approach was followed in the reported work by Tan et al.^36^ to pattern PTFE membrane via thiol-ene to fabricate an organ-on-a-chip model. Nevertheless, this approach is only suitable for hydrophilic and porous membranes that could absorb photo-crosslinkable glues. Some plastic membranes could attach using heat-based methods^37,38^. However, heat would not be used to assemble membranes due to the thermoset properties of photo-crosslinkable resins^39^ such as Dental LT.

SLA or DLP 3D printers have much higher resolution than needed for these inserts. The only problematic parameter is the high aspects ratio combined with relatively thin walls of the insert. Here we used 500 µm walls, but thinner walls would make it possible to fit more layers into a given well size (Fig. 1). However, that will challenge the structural integrity of the inserts unless other materials than the brittle Dental LT can be employed. The most significant issue with fitting many layers of inserts is the capillary effects between the walls and bubble capture. Empirically, we have found that 750 µm distance to be acceptable using the given materials and mediums. A more hydrophobic material would decrease the capillary effects and allow for more layers in each well size. However, this will likely increase the difficulties for liquid handling to avoid e.g., bubbles being trapped in smaller diameter wells. From the organ modelling point of view, many layers may not be needed. For instance, to model the path from the intestine to the brain would require three layers: one for the intestine/blood barrier, one for the liver and one for the blood–brain barrier where the brain model is located at the bottom of the well. Three layers could fit using the current design in 24- to 48-well plates, but with material improvements suppressing capillary effects, a 96-well plate could be utilized (Fig. 1).

Distributed fabrications of customized inserts allow for any adjustment such as the distance between the layers, the number of layers and the material and sizes of the bioactive area in the bottom of each insert. For distributed fabrication to be widespread, it must be cost-efficient and accessible. Many easy-to-use 3D printers can be hosted in any bio-lab as these printers are intended for custom dentistry applications. The costs of 3D SLA and DLP printers vary a lot depending on one vendor to another ($2000–3000)^40^. Low-cost printers < $1000 are available in the market, but they are still not convincingly tested in biocompatibility of printing resins for cell culture applications. Especially, dental resin formulations such as Dental LT resin from Formlabs are biocompatible but still need to be tested for individual cellular experiments. Moreover, alternative resin formulations and post-processing methods^14^ could be explored for biocompatibilities of 3D-printed inserts for customization purposes. A commercial 12-well Transwell inserts cost 8€/ insert (Supplementary Table S1). By contrast, the material cost of an insert 3D-printed with Dental LT resin is about 7 times less despite the costs of the biocompatible resin (Supplementary Table S1). Post-processing takes approximately 30 min of hands-on time for a full plate of inserts, making the price for distributed fabrication competitive.

The bottom defines how the cells will react. As apparent, the Dental LT resin is biocompatible but not bioactive, and cells do not grow directly on it without surface coating with bioactive motives^5,41,42^ (Fig. 4). Bioactive and bio-passive patterning can be achieved by controlling these cell adhesion areas. The dip-casting procedure can produce cm^2^ large hydrogel films^16^. However, these hydrogels are flexible, which can negatively affect barrier function^5^. Therefore, many smaller apertures (0.5–4 mm in diameter) were tested here. However, the geometry can be designed freely (Supplementary Fig. S2). While not crucial for the barrier function explored, the shape of the cell culture area can align, for instance, muscle cells^27^.

To conclude, we showed that 3D-printed inserts are a solid alternative to commercialized inserts that cannot provide the freedom to customize inserts and provide layers of the inserts. It opens the door for any scientists to design their own culture ware more imaginatively and tailored to the experiment. We also reported that dip-casting could offer a straightforward method to create hydrogel membranes, and control experimental conditions would improve the standardization of the method and membrane fabrication efficiency.

## Methods

### Fabrication of 3D-printed inserts

3D-printed inserts with different formats and dimensions (Fig. 1c) were designed for tissue-culture-treated (TCT) well plates (Nunc, Thermo Fisher, Slangerup, DK) using Fusion 360 (Autodesk, California, USA) and exported as .STL format files. The print files were processed by adding external supports (with 0.5 density and 0.4 mm touchpoint size and mini rafts with 0.5 mm thickness) and converted to .FORM files in PreForm (v.3.11.0, Formlabs, Somerville, Massachusetts, USA) before printing by a Form 3B 3D printer (Formlabs). Inserts were printed in Dental LT v1 resin (Formlabs) with a layer thickness of 0.1 mm. After printing, inserts were washed in the isopropyl alcohol (IPA) tank (Formlabs) and the support structures were cut off. All prints were subsequently cleaned twice in fresh IPA for 1 h, respectively. After washing, inserts were air-dried and post-cured in the Form Cure UV-oven (Formlabs) for 2 h at 60 °C. After the post-curing step, inserts were submerged in sterile water with a 30 mL resin to 1 L water ratio for a day. Once the prints were air dry, a further 15 min UV exposed for each side for sterilization was performed using Asiga Flash UV Curing Chamber (New South Wales, AUS) under the laminar flow biosafety level 2 (BSL-2) cabinet.

### Assembling synthetic and hydrogel membrane

Hydrophilic Polytetrafluoroethylene (PTFE) membrane (BioPore Membrane Filter, with a pore size of 0.4 µm, Millipore®, Sigma-Aldrich, Missouri, USA) was assembled on the bottom of the 3D-printed insert and referred to as Filter inserts). Dental LT was applied to the insert walls and punched out PTFE membrane discs was affixed. Afterwards, the membrane was fixed towards the wall of inserts using a printed cylindrical ring that also stretched the filter. It was post-cured in a Form Cure UV-oven (Formlabs) for 20 min at 60 °C. Sequential IPA washing steps and UV post-curing were conducted as described above.

The gelatin (48723, Sigma-Aldrich) was mixed with 5 mL phosphate-buffered saline (PBS) (D8537, Sigma, St Louis, USA) and heated to 37 °C. The solution was adjusted to pH 7 by titrating with a 10 M NaOH. For a 5 mL 10% solution, approximately 3.7 µL of 10 NaOH was added. For 5 mL 15% and 20% gelatin solutions, the corresponding values were 5.7 and 7.7 µL. Chloroform was added (0.5% v/v) to sterilize the solution. These standardized volumes made it possible to mix all components and place the tubes a in a 37 °C water bath until the gelatin was fully dissolved to obtain a sterile working solution with the correct pH. Once dissolved, gelatin solutions were poured to a pre-heated sterile petri dish (Nunc, Thermo Fisher Scientific, USA) on the homemade hot plate (Supplementary Fig. S3) set at 50 °C under the laminar flow biosafety cabinet. Note, the LAF bench requires an exhaust into free air as the chloroform will evaporate quickly. Sterile 3D-printed inserts without PTFE membrane were immersed in the gelatin solution for 5 s until the bottom was covered with hydrogel liquid. The inserts were fully withdrawn and pressed on a sterile pre-warmed petri dish lid on the homemade Peltier unit at 50 °C to form a thin hydrogel membrane by removing the extra hydrogel liquid. After dip-casting the membranes, inserts were placed to the cell culture plate and placed at 4 °C for at least 20 min to thermally (reversible) crosslink the gelatin. In the meantime, 15 U/mL microbial Transglutaminase (mTG) solution was prepared by mixing mTG (1002, 100 U/g, ACTIVA® TI, Ajinomoto Food Ingredients LLC, Illinois, USA) with PBS. The mTG solution was vortexed until the mTG powder was fully dissolved and filtered through a 0.45 µm sterile syringe filter (Avantor, VWR, USA). After the reversible crosslinking of the gelatin membranes, the mTG solution was added to wells with the amount to cover insert well surfaces (for 96-well: 50 µL; for 48-well: 100 µL; for 24-well: 200 µL). The plates were placed in an incubator at 37 °C for 1 h to crosslink membranes irreversibly. After crosslinking, the sterile hydrogel membranes were washed in PBS for 10 min three times. All inserts with gelatin membrane (Gelatin-inserts) were either used for cell culture experiments directly or stored in the fridge for max. 2 weeks or dried overnight in the incubator and stored at room temperature for maximum 1 month prior to use.

### Mass transport through membranes

The permeability of 96-well strip inserts was tested by mass transport studies of Fluorescein sodium salt (F6377, Sigma-Aldrich). Inserts were placed in a 96-well plate and, 225 µL of pre-heated (to 37 °C) PBS was added to the basolateral side, and 75 µL of 50 µM Fluorescein sodium salt was added to the apical side in each insert. The transport study was performed at 37 °C with 100 rounds per minute (rpm) shaking under dark conditions for 2 h. After the incubation, samples of 50 µL were transferred to a 96-well plate to analyze their Relative Fluorescence Units (RFU) emission at an excitation wavelength of 460 nm and emission wavelength of 515 nm (Spark® multimode microplate reader, TECAN, Männedorf, CH). A standard curve was obtained by evaluating RFU of fluorescein sodium salt in different concentrations from 50 µM to 0 µM, and RFU data was collected and analyzed using GraphPad Prism (version 9.0.0 for macOS, GraphPad Software, La Jolla California USA, www.graphpad.com).

### Mechanical testing of hydrogel membranes

Gelatin hydrogel discs were prepared using the sandwich casting method. Two polymethyl methacrylate (PMMA) slides with a thickness of 3 mm were coated with 5% (w/v) PVA (Polyvinyl alcohol)–EtOH (ethanol) solution used as a mold released agent and air-dried. Afterwards, two 1 mm thick-PMMA slides were glued on two pairs of 3 mm-thick PMMA slides (base) with PVA solution. 500 µL gelatin solution was cast on one of the base slides, and the other base slide was closed on top of the other pair. Then, gelatin discs with 1 mm thickness were crosslinked in the fridge at 4 °C. After crosslinking, the gelatin discs were carefully removed by flushing mTG solution between the base slides and incubated in mTG loaded petri dish for 1 h in the incubator at 37 °C. Irreversibly crosslinked gelatin discs were washed with PBS twice and stored in a closed petri dish with PBS to avoid dehydration. Rheological properties were characterized using a rheometer (TA Instruments, DHR 20, New Castle, Delaware, USA) equipped with a 20 mm diameter cylinder with parallel plate geometry and a gap of 1 mm. Tests in gelatin discs were performed at 37 °C. Manufactured samples were loaded on the lower Peltier plate of the rheometer and trimmed to adjust the shape to the upper parallel plate geometry. First, a frequency sweep test was performed from 200 rad/s to 1 rad/s at the strain of 0.01% to calibrate the instrument. Then, the amplitude/strain sweep test was performed from 0.2 to 200% at 1 rad/s, and the results were used to evaluate material properties. Finally, time sweep up to 1000 s (about 16 and a half minutes), at 5% strain and 1 rad/s to ensure that the material was stable in the estimated time. Stiffness and elasticity of the developed hydrogels were assessed from shear modulus, and Young’s modulus of each disc was obtained as previously described^16^. The shear modulus or complex modulus (G^∗^) was calculated by:

1 $${G}^{*} = \sqrt{{G}^{^{\prime}2}+{G}^{^{\prime}{^{\prime}}2}}$$

where, G’ is the storage modulus, and G” is the loss modulus.

Then, Young’s modulus (E) was obtained as:

2 $$E=2{G}^{*}(1+ \mu )$$

where, μ is the Poisson’s ratio, assumed to be 0.5 as the hydrogels are considered incompressible materials.

### Thickness measurement of hydrogel membranes

For measurement of gelatin membrane thickness, fluorescence intensity analysis was performed. Fluorescent gelatin was prepared by mixing 15% (w/v) gelatin powder in 1 µg/mL Fluorescein sodium salt (F6377, Sigma-Aldrich)—PBS solution. First, 6 liquid coupled slides were created by using the similar sandwich method described before to form the standard curve of fluorescence intensity of different heights provided by stacking coverslips (150 µm thick) onto a 1 mm thick glass microscope slide (base) and 500 µL fluorescence gelatin was pipetted onto the base slide and covered with the other pair of the base slide. Inserts (96- and 48-well format strips) were fabricated as described above and placed onto a base slide (Avantor, VWR, USA). Gelatin membranes were imaged with Zeiss™ AxioObserver Z1 epifluorescence microscope (Carl Zeiss MicroImaging GmbH, Gottingen, Germany). All images (n = 5) were obtained at an emission wavelength range of 495 nm to 517 nm by a LED (Light-emitting Diode) laser with filter set 38 HE (Carl Zeiss MicroImaging GmbH) and within 150–900 µm thickness range. All the .TIF files were processed with Zeiss Zen Blue 3.4 Lite Digital Imaging Software (version 3.4.91.00000, Carl Zeiss Microscopy GmbH) to correlate membrane height according to fluorescence intensity.

### Swelling ratio of hydrogel membranes

For determination of thickness change of hydrogel membranes, 15% (w/v) Gelatin membranes were assembled to 48-well format Gelatin-inserts as mentioned above. Membranes were punched with 3 mm diameter biopsy skin punch (Acu-Punch®, Acuderm Inc., USA)) as freshly crosslinked (wet, n = 5), and swollen (n = 5) in PBS for 1 h at 37 °C to mimic equilibrate status of membranes and dehydrated overnight at room temperature (dry, n = 5). The thickness of punched membrane discs was measured by using a micrometer screw (Mitutoyo, JP) and the swelling ratio (SF_v_) was calculated by deriving the equation below^43^ (diameter was taken constant due to the dry, wet, and swollen membranes were taken out of the inserts using the puncher:

3 $${SF}_{v}= \frac{\pi {r}_{f}^{2}{h}_{f}}{\pi {r}_{0}^{2}{h}_{0}}= \frac{{({d}_{t}/2)}^{2}{h}_{f}}{{({d}_{0}/2)}^{2}{h}_{0}}= \frac{{-\!\!\!d}_{-\!\!\!t}^{-\!\!\!2}{h}_{f}}{{-\!\!\!d}_{-\!\!\!0}^{-\!\!\!2}{h}_{0}}= \frac{{h}_{f}}{{h}_{0}}$$

where, r_f_ is the final radius (µm), h_f_ is the final height (µm), r_0_ is the initial radius (µm), h_0_ is the initial height (µm), d_f_ is the final diameter (µm), d_0_ is the initial diameter (µm).

### Cell types and culture conditions

Human epithelial colon carcinoma cells (Caco-2, passage 55–65, 09042001, European Collection of Authenticated Cell Cultures (ECACC), Salisbury, UK) were cultured in T-75 cell culture flasks (Sarstedt, Nümbrecht, Germany) in High-glucose Dulbecco's DMEM medium (Sigma-Aldrich) with 10% (v/v) fetal bovine serum (FBS, Hyclone™, CA), 1% (v/v) non-essential amino acids (NEAA, Gibco, Fisher Scientific, Slangerup, Denmark), and penicillin (100 U/mL)-streptomycin (100 µg/mL) (P/S, Sigma-Aldrich). The Caco-2 cells were seeded on hydrogel membranes at a density of 80,000 cells/cm^2^ and incubated for 3 weeks before analyses. Human umbilical vein endothelial cells (HUVEC, passage 5–9, Cell Applications Inc., California, USA) were cultured in endothelial culture growth medium (ECGM, Cell Applications) with 10% (v/v) FBS and 1% (v/v) P/S. The HUVEC cell cultures were seeded on gelatin micro-membranes and Matrigel-coated PTFE membranes at a density of 100,000 cells/cm^2^ and incubated for up to 1 week before analyzes. Cells were split with trypsin-ethylenediaminetetraacetic acid (EDTA) for 3–5 min upon ~ 90% confluency. After use, the gelatin membranes could be dissolved using trypsin–EDTA to reuse the 3D-printed parts. Mouse intestinal organoids were cultured according to the protocol from StemCell Technologies (Cambridge, UK). Intestinal crypts were thawed, centrifugated and re-suspended in Matrigel (Corning, New York, USA) and transferred into 24-well PTFE membrane assembled insert plates. After polymerization, IntestiCult mouse organoid growth medium (StemCell Technologies) supplemented with 1% (v/v) P/S was overlaid on the gel in each well. All cell lines and organoids were maintained in an incubator (37 °C, 100% Humidity, 5% CO_2_) with the culture medium replaced every 2 days.

### F-actin, nuclei, live/dead stains

For F-actin (Alexa Fluor™ 594 Phalloidin, Invitrogen) and Hoechst 33342 (nuclei, Invitrogen™, Thermo Fisher) staining, samples were washed for 15 min in PBS followed by fixation in 2% (v/v) PFA in PBS for 2 min and fixation in 4% (v/v) PFA in PBS for 13 min at room temperature. PFA was aspirated from the samples and washed three times in PBS. Then, the samples were incubated with the stain (5 µL phalloidin and 1 µg/mL Hoechst 33342 in PBS diluted in 200 µL PBS with 1% (v/v) bovine serum albumin (BSA, Sigma)) for 20 min at room temperature. The samples were washed three times in PBS and left in PBS.

For live/dead staining (LIVE/DEAD™ Viability/Cytotoxicity Kit, L3224, Invitrogen™, Thermo Fisher) was used. Samples were washed three times in PBS and stained in 500 µL of a solution with Ethidium homodimer-1 (8 nM, EthD-1) and Calcein AM (4 mM) for 1 h at room temperature. The samples were washed in PBS two times and then kept in PBS to keep the hydrogel growth-matrices moisturized.

### Microscopy imaging

Phase-contrast bright-field micrographs were obtained using a Zeiss Primovert microscope (Carl Zeiss MicroImaging GmbH, Gottingen, Germany) with the following objective: Plan-Achromat 4x/0.10 while staining images were acquired with Zeiss™ AxioObserver Z1 epifluorescence microscope (Carl Zeiss MicroImaging GmbH) with the following objectives: EC Epiplan-NEOFLUAR 5x/0.16 Ph1 M27: EC Epiplan-NEOFLUAR 10x/0.3 Ph1, LD Epiplan-NEOFLUAR 20x/0.4 Korr M27. The obtained images were fitted with scale bars and were processed in Zeiss Zen Blue 3.4 Lite Digital Imaging Software (v. 3.4.91.00000, Carl Zeiss Microscopy GmbH) and ImageJ/Fiji^44^. Surface area analysis of organoids were performed using the macro; OrgM^45^ on the ImageJ/Fiji.

### Transport of lucifer yellow

Single inserts were placed in a 96-well plate and 225 µL (well compartment) of pre-heated (to 37 °C)) HBSS transport buffer (HBSS (1x), Sodium bicarbonate (0.0375% w/v), HEPES (10 mM), BSA (0.05% w/v, pH 7.4) was added to the basolateral side and 75 µL of 60 µM lucifer yellow was added to the apical side in each insert. The transport study was performed at 37 °C with 100 rounds per minute (rpm) shaking under dark conditions for 2 h for Caco-2 cells. After the transport experiment, the 100 µL samples (from 1st insert and the well compartments), 50 µL samples (from 2nd insert compartment) and 50 µL HBSS transport buffer were transferred to a 96-well plate to analyze their Relative Fluorescence Units (RFU) emission at an excitation wavelength of 428 nm and measuring emission at 536 nm. The permeability coefficients (Pc, nm/s) were calculated by the equation:

4 $$Pc=({V}_{r}\times {C}_{f})/({C}_{i}\times A\times t)$$

where, Pc is the permeability coefficient (nm/s), V_r_ is the receiver volume in mL, A is the membrane growth area in cm^2^, C_i_ is the initial apical concentration (µM), C_f_ is the final receiver concentration (µM), t is the assay time in seconds.

### Statistical analysis

The data are presented as the sample sizes (n), means, standard deviations (SDs). Calculations were done using Microsoft Excel (Version 2016, Microsoft Office, Seattle, Washington) and data were analyzed using GraphPad Prism (version 9.0.0 for macOS, GraphPad Software, La Jolla California USA, www.graphpad.com). Saphiro–Wilk for normality testing of multiple comparisons. Data was analyzed by ordinary one-way ANOVA or 2way ANOVA (α = 0.05) with post-hoc Tukey’s multiple comparison tests for equality of means among groups in membrane characterization, lucifer yellow transport of stacked co-culture and biocompatibility of organoids experiments. P-values were obtained using Bartlett’s corrections and determined significant differences when p-value < 0.05. F-test for Welch’s t-test was performed to compare variances of membrane thicknesses. The significant differences are highlighted with symbols on figures.

## Supplementary Information

Supplementary Information.

## Data Availability

STL files of 3D-printed designs are available from the authors upon request. A list of links to repository will be hosting the respective. STL is provided below: https://www.thingiverse.com/asliaybikedogan/collections/customized-3d-printed-inserts.
