# Author Correction: Customized 3D-printed stackable cell culture inserts tailored with bioactive membranes

**DOI:** 10.1038/s41598-022-09955-7

**Published:** 2022-04-06

**Authors:** Asli Aybike Dogan, Martin Dufva

**Affiliations:** Department of Health Technology, Technical University of Denmark, 2800 Kgs. Lyngby, Denmark

Correction to: *Scientific Reports* 10.1038/s41598-022-07739-7, published online 07 March 2022

The original version of this Article contained an error in Figure 5 where the labels “PTFE” and “Gelatin” were interchanged. The original Figure 5 and accompanying legend appear below.

**Figure 5:**
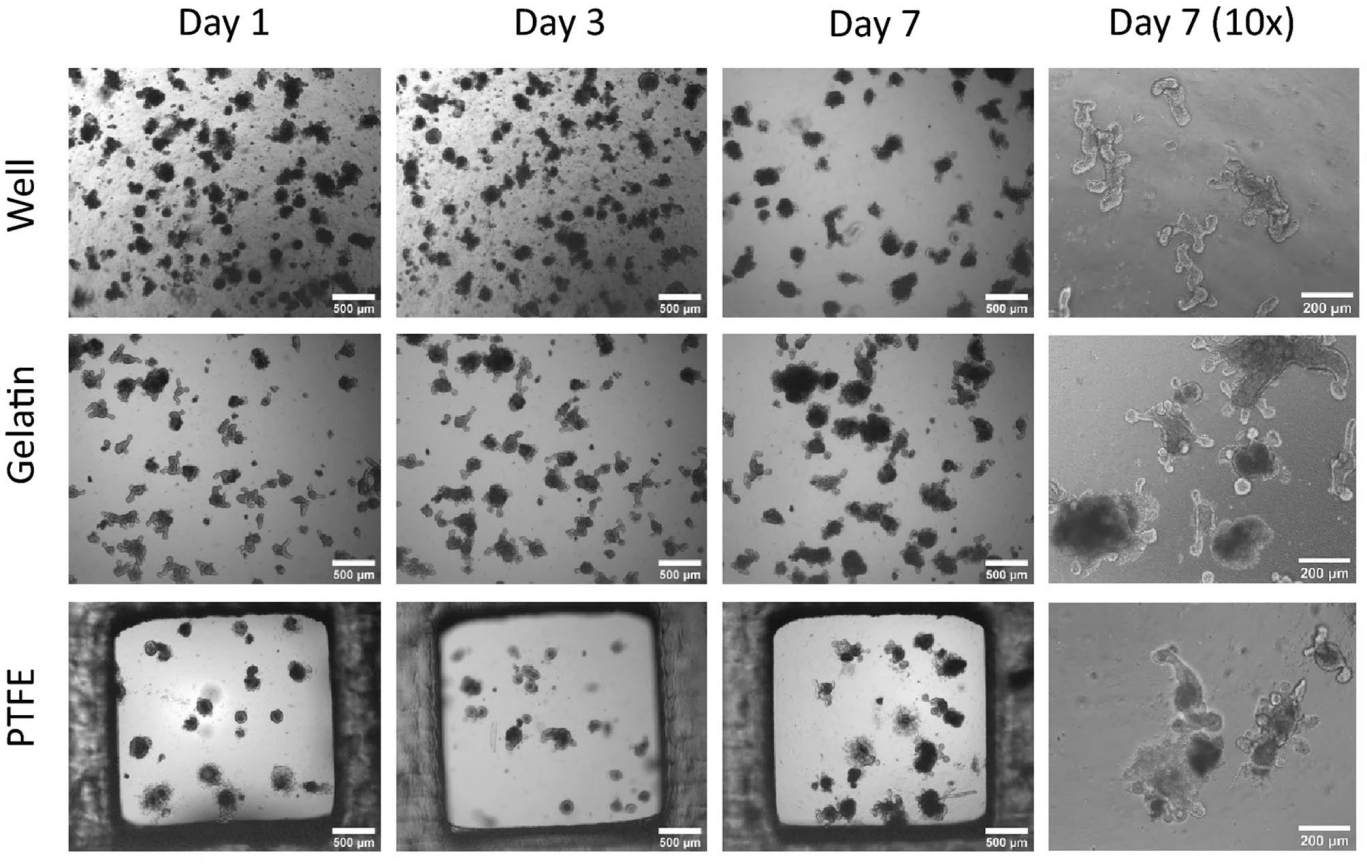
Representative light microscopy images of mouse intestinal organoids in Matrigel dome on hydrophilic PTFE filters and Gelatin membrane. Images (n = 3) were taken during their culture for 7 days in the insert plate wells with a light microscope (scalebar 500 µm for 4 × magnification; scalebar: 200 µm for the 10 × magnification.

The original Article has been corrected.

