# Supplementary material for: Customized 3D-printed stackable cell culture inserts tailored with bioactive membranes
Source: Sci Rep. 2022 Mar 7;12:3694. doi: 10.1038/s41598-022-07739-7 (PMC8901659; doi:10.1038/s41598-022-07739-7)
Supplement: Supplementary file 1 — Supplementary Information. [file 41598_2022_7739_MOESM1_ESM.docx]

# **Customized 3D-printed stackable cell culture inserts tailored with bioactive membranes**

# Asli Aybike Dogan^1^, Martin Dufva^1^, *

1Dept. of Health Technology, Technical University of Denmark, 2800 Kgs. Lyngby, Denmark.

*[dufva@dtu.dk](mailto:dufva@dtu.dk)

**Supporting Information**

**Table S1.** The source, printing time and cost of materials for fabricating inserts comparing the cost of commercialized peers.


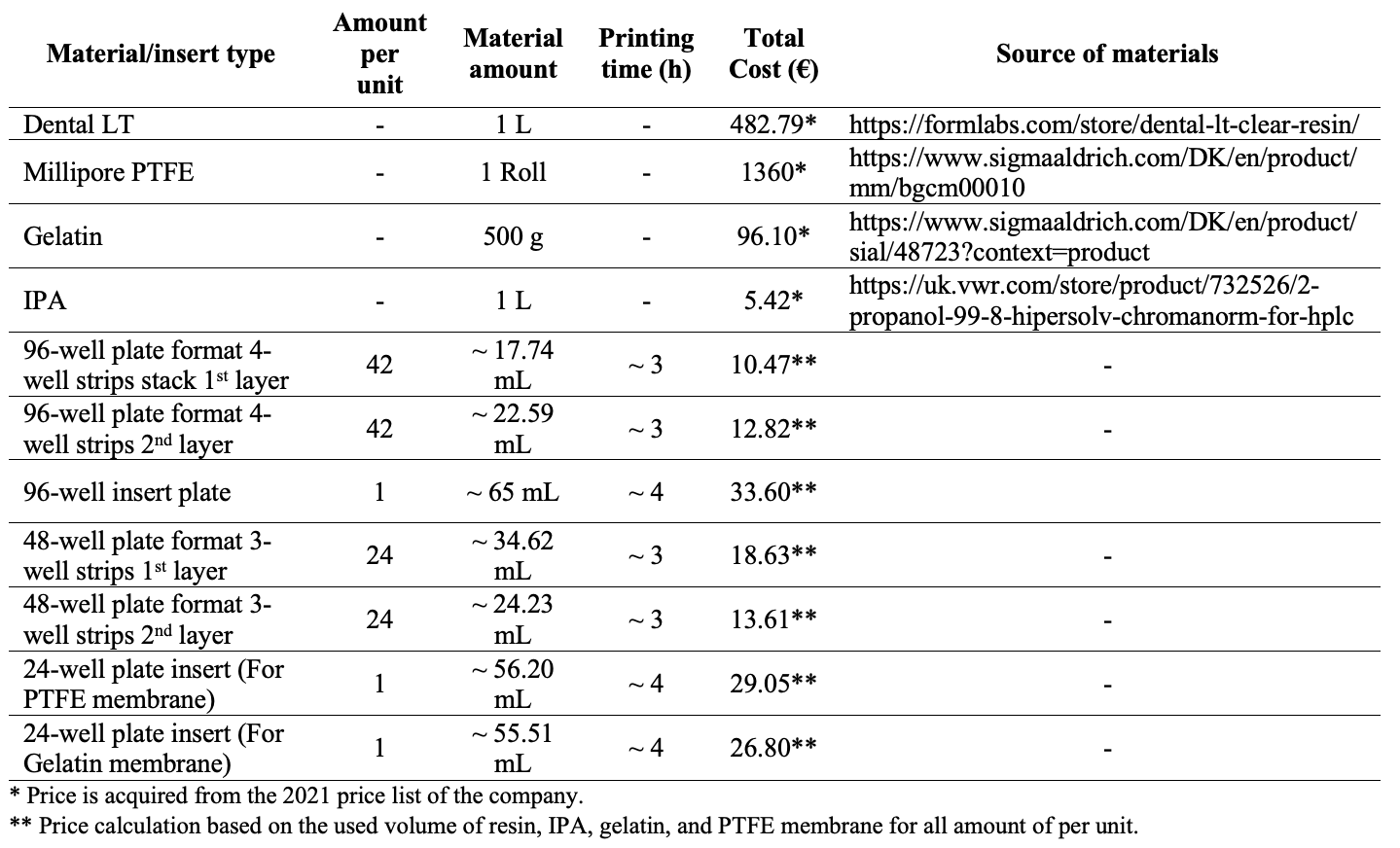


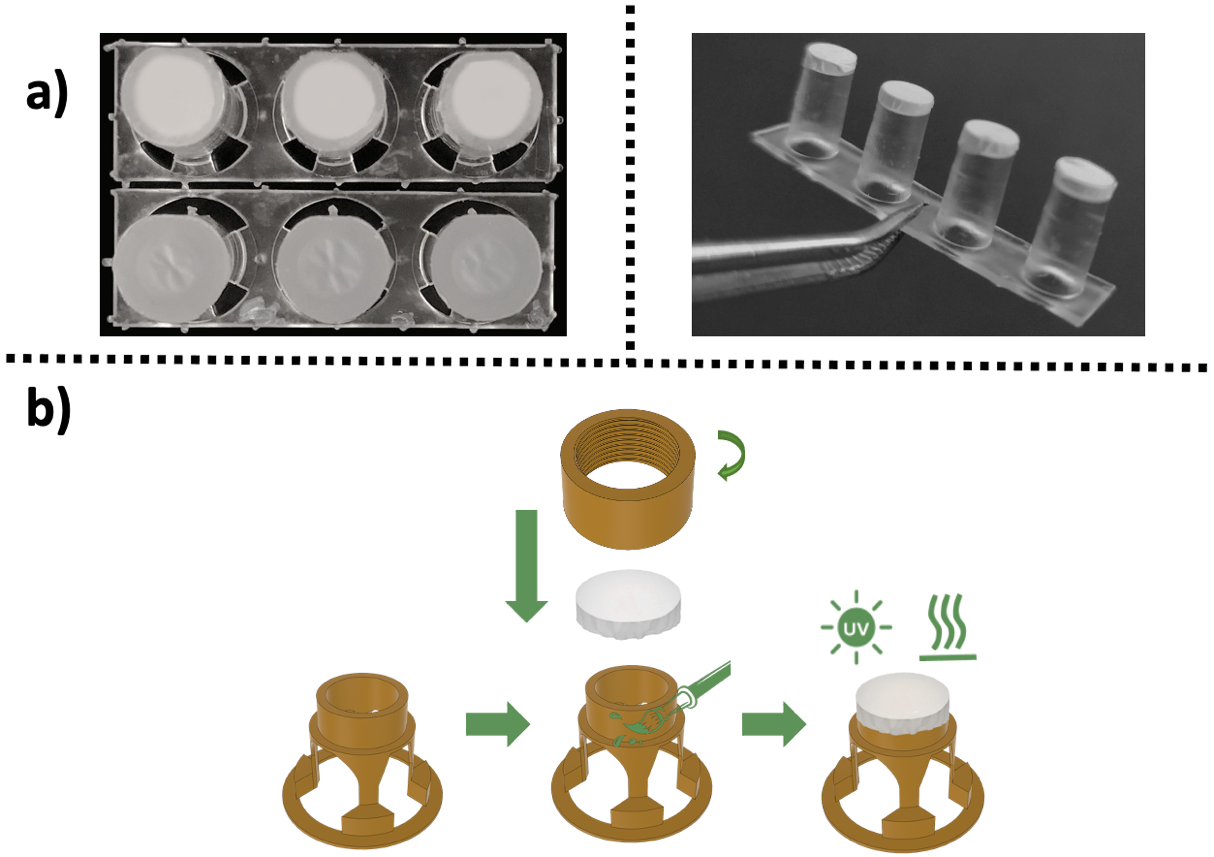


**Figure S1**. **a)** ***Left:*** 48-well format strip inserts glued from off-set of apertures (***top:*** partially clogged PTFE membrane; ***bottom:*** Clogged PTFE membrane (***white area:*** view of dry hydrophilic PTFE membrane; ***shaded area:*** view of wet/glued hydrophilic PTFE membrane). ***Right:*** 96-well format strip insert glued from side walls of wells). **b)** Illustration of hydrophilic PTFE membrane glued with Dental LT resin to the 3D-printed inserts.


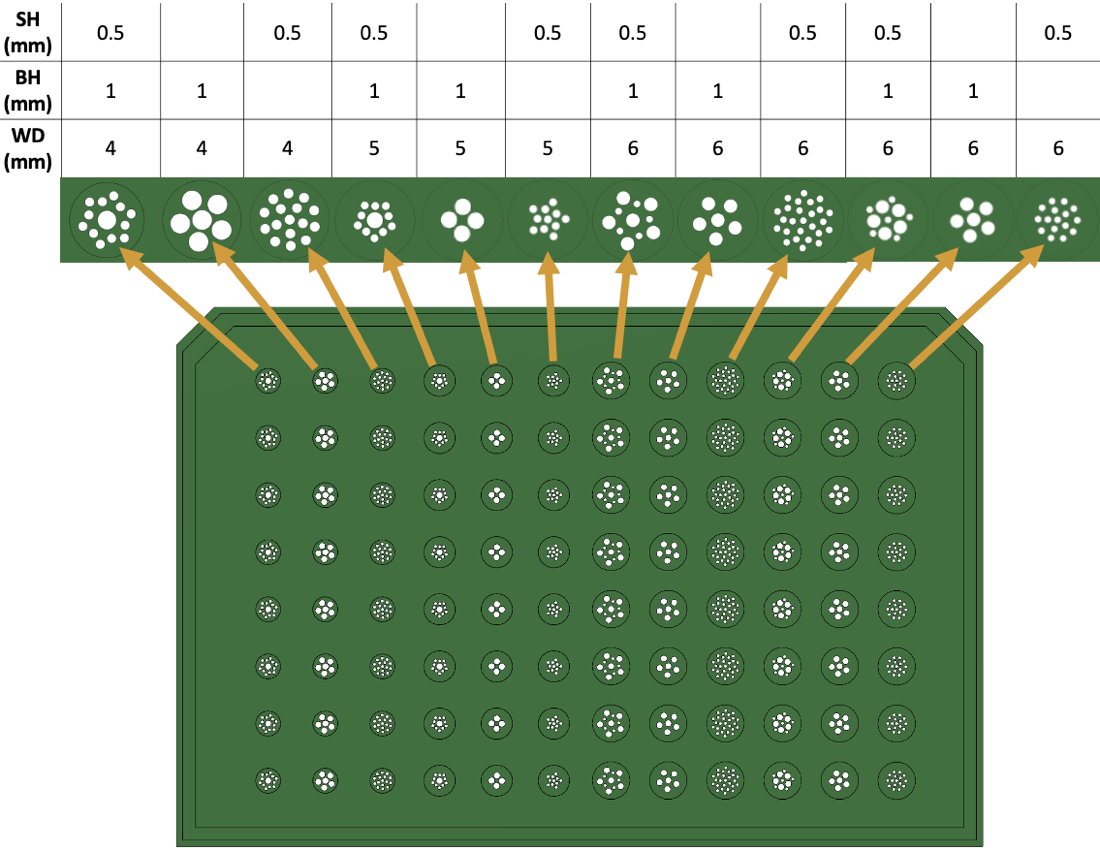


**Figure S2.** Illustration of 96-well format insert plate with different aperture patterns. *SH:* Small holes (Ø=0.5 mm), *BH:* Big holes (Ø=1 mm), *WD:* Well diameter (Ø=4-6 mm).


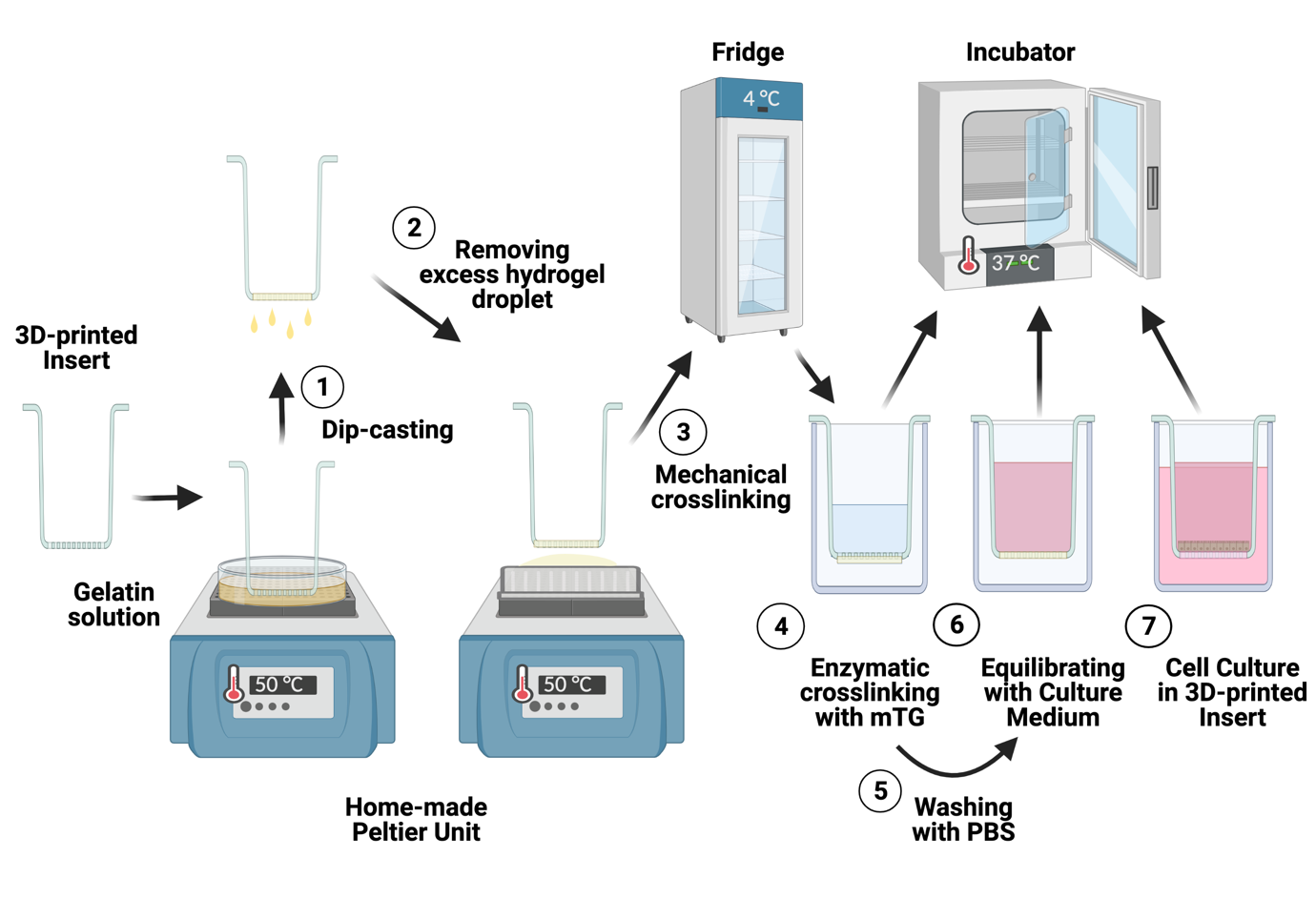


**Figure S3.** Illustrative outline of **1-2)** the dip-casting method were performed at approx. 37 °C using a home-made Peltier unit at 50 °C for Gelatin hydrogel membrane assembling to the 3D-printed inserts platforms. **3)** Gelatin membranes were mechanically crosslinked at 4 °C for at least 20 mins. Then, **4)** enzymatically crosslinked at 37 °C for 1 h. **5-7)** Membranes were washed and equilibrating with culture media before cell culture experiments. *Created with BioRender.com*


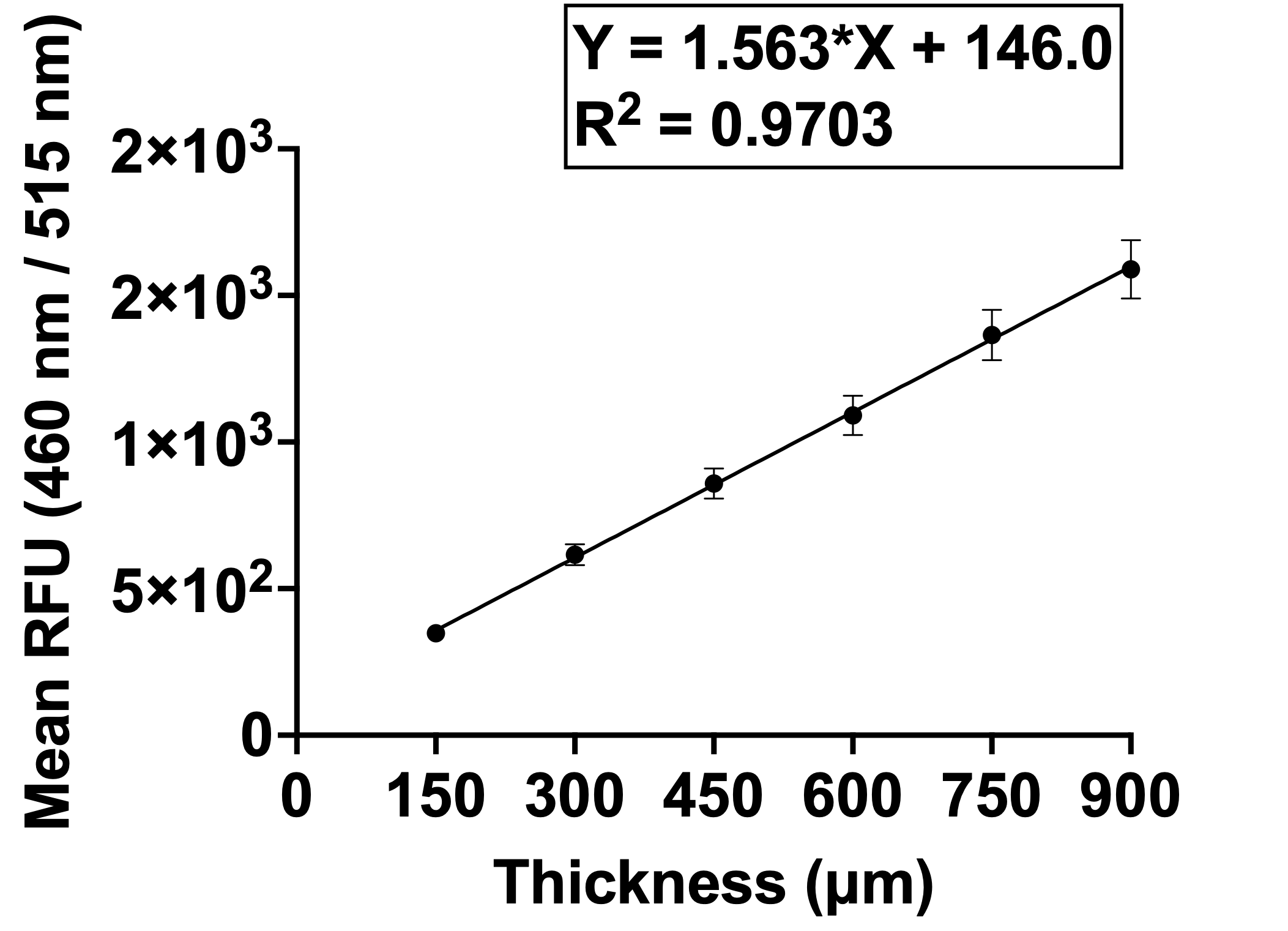

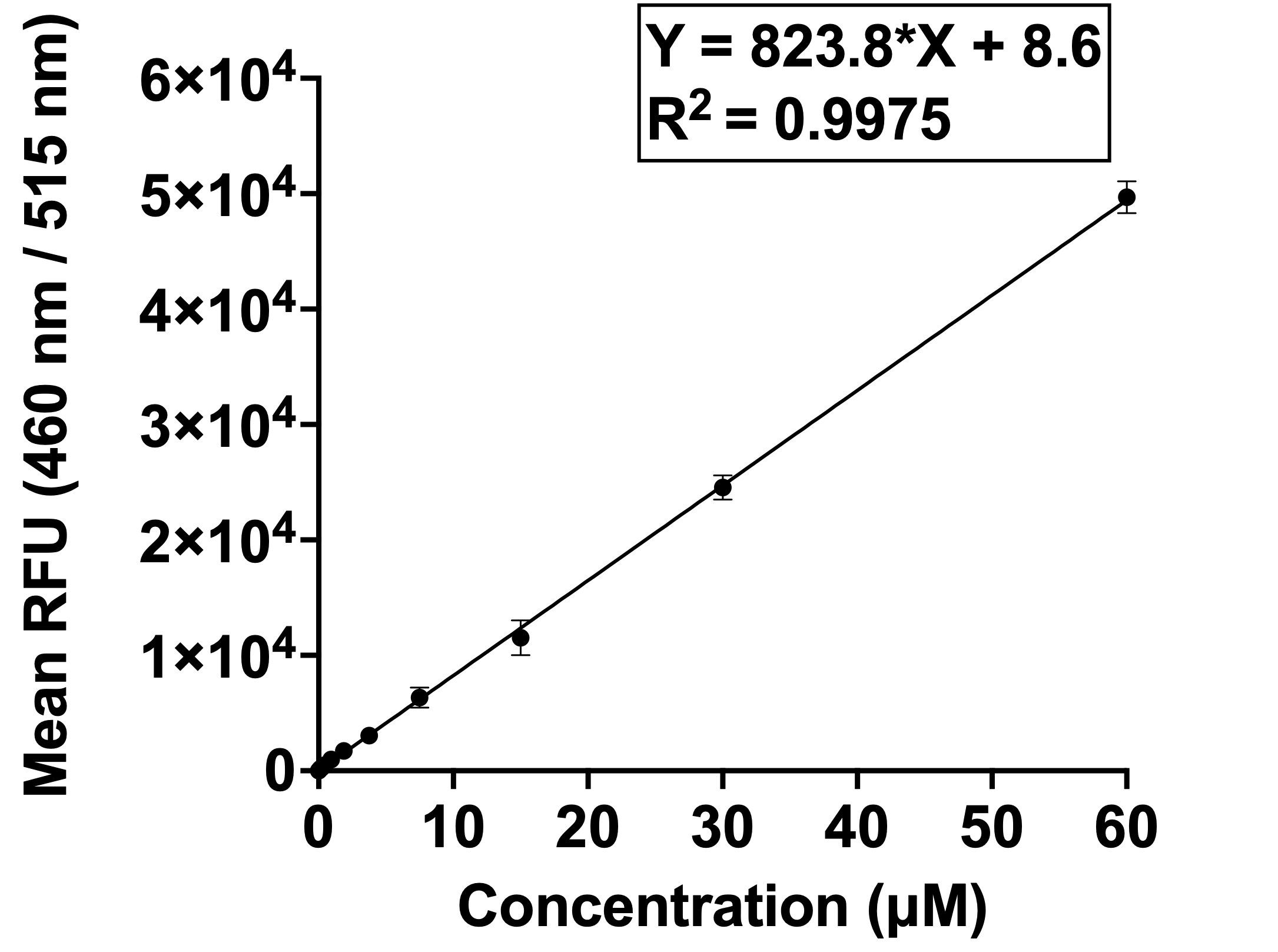


**Figure S4.** ***Left:*** Standard curve of fluorescein intensity for Gelatin membrane thickness measurement. ***Right:*** Standard curve of Fluorescein (0-60 µM) for Gelatin membrane permeability evaluation.


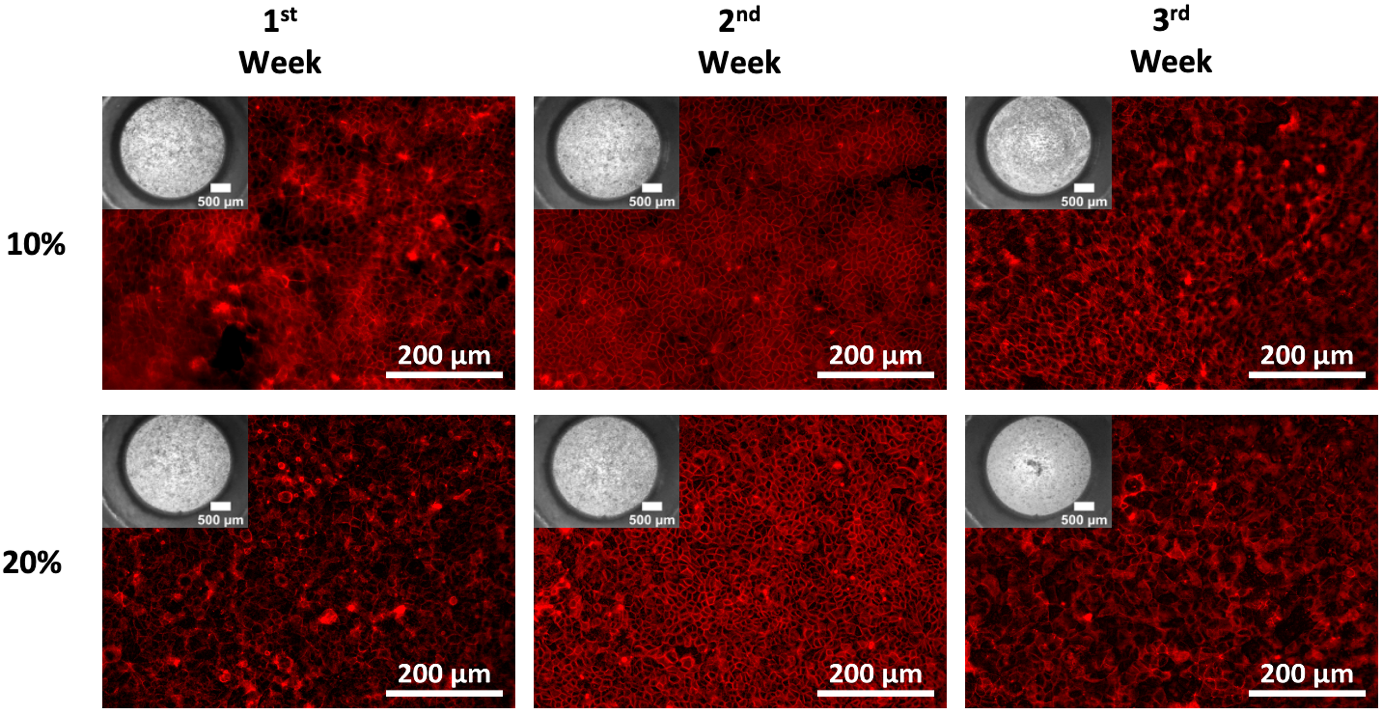


**Figure S5.** Biological validation of 96-well inserts. **a)** Growth of Caco-2 cells on the 10% and 20% (w/v) gelatin membranes over three weeks. Insert apertures at the bottom are shown at upper panels of fluorescence images (the magnification: 4x; scale bars: 500 µm), and the F-actin (red) stain of the cells on the hydrogel membranes (scale bars: 200 µm; the magnification was 20x).


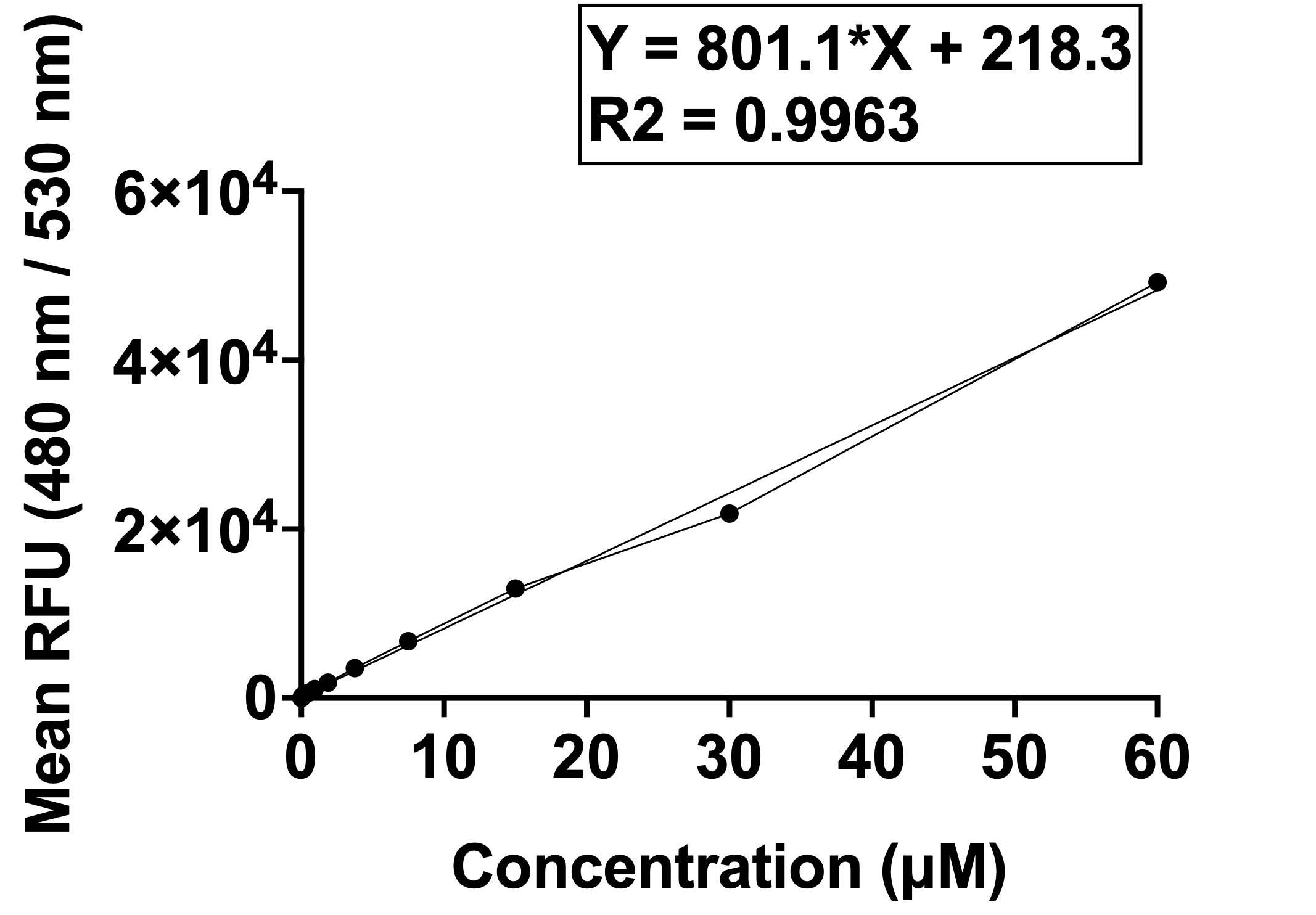


**Figure S6.** Standard curve of Lucifer Yellow (0-60 µM) for calculation of permeability coefficient to evaluate barrier functions of Caco-2 cells.


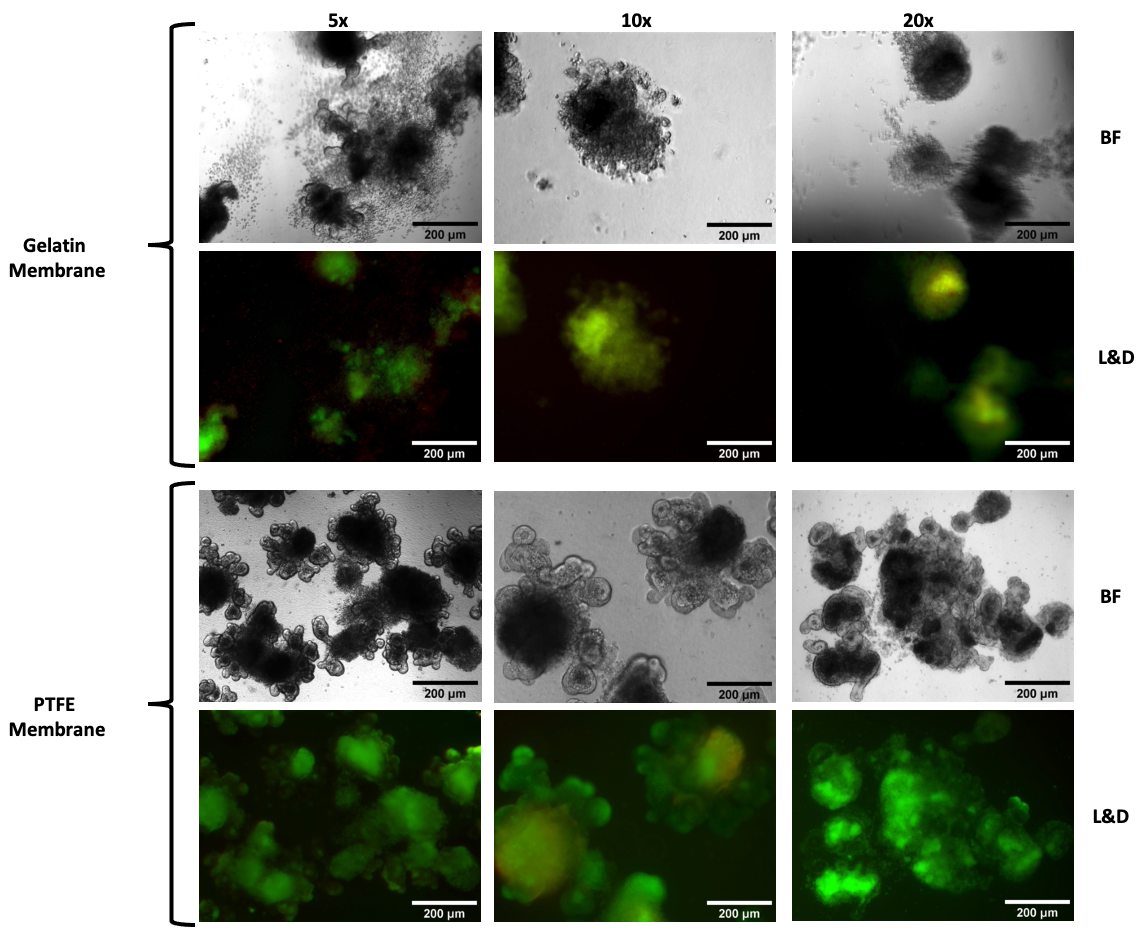


**Figure S7.** Brightfield (BF) and Live & Dead (L&D) staining photos of mouse intestinal organoids on day 10. Organoids in 24-well format square grid patterned Gelatin- and filter-insert plates. Scalebar: 200 µm, the magnification: 5x-10x-20x)
